# Involvement of MIF in Basement Membrane Damage in Chronically UVB-Exposed Skin in Mice

**DOI:** 10.1371/journal.pone.0089569

**Published:** 2014-02-21

**Authors:** Yoko Yoshihisa, Osamu Norisugi, Kenji Matsunaga, Jun Nishihira, Tadamichi Shimizu

**Affiliations:** 1 Department of Dermatology, Graduate School of Medicine and Pharmaceutical Sciences, University of Toyama, Toyama, Japan; 2 Department of Medical Information, Hokkaido Information University, Ebetsu, Japan; Columbia University Medical Center, United States of America

## Abstract

Solar ultraviolet (UV) B radiation is known to induce matrix metalloproteinases (MMPs) that degrade collagen in the basement membrane. Macrophage migration inhibitory factor (MIF) is a pluripotent cytokine that plays an essential role in the pathophysiology of skin inflammation induced by UV irradiation. This study examined the effects of MIF on basement membrane damage following chronic UVB irradiation in mice. The back skin of MIF transgenic (Tg) and wild-type (WT) mice was exposed to UVB three times a week for 10 weeks. There was a decrease in intact protein levels of type IV collagen and increased basement membrane damage in the exposed skin of the MIF Tg mice compared to that observed in the WT mice. Moreover, the skin of the MIF Tg mice exhibited higher MIF, MMP-2 and MMP-9 expression and protein levels than those observed in the WT mice. We also found that chronic UVB exposure in MIF Tg mice resulted in higher levels of neutrophil infiltration in the dermis compared with that observed in the WT mice. *In vitro* experiments revealed that MIF induced increases in the MMPs expression, including that of MMP-9 in keratinocytes and MMP-2 in fibroblasts. Cultured neutrophils also secreted MMP-9 stimulated by MIF. Therefore, MIF-mediated basement membrane damage occurs primarily through MMPs activation and neutrophil influx in murine skin following chronic UVB irradiation.

## Introduction

Exposure to ultraviolet (UV) radiation leads to various acute deleterious cutaneous effects, including sunburn and immunosuppression. One of the detrimental long-term effects of UV exposure is cutaneous photoaging. Photoaged skin is biochemically characterized by a predominance of abnormal elastic fibers in the dermis and a dramatic decrease in certain types of collagen. Interstitial collagens, the major structural components of the dermis, are particularly reduced in UV-irradiated actinically damaged skin [1]–[3].There are several morphological and biochemical indicators suggesting that the amount and normal structure of collagen type I is reduced in UV damaged skin [4].

UV-exposed skin cells, including keratinocytes and fibroblasts, contain large quantities of active collagenase [5]. Matrix metalloproteinases (MMPs) are a family of secreted and transmembrane proteins capable of digesting the extracellular matrix and basement membrane components under physiological conditions. MMPs are induced by various extracellular stimuli such as UV radiation, growth factors and cytokines [6]–[8]. The MMP-1 expression plays a major role in the photoaging process induced by degeneration of type I collagen in the dermis [4], [9]. Other MMPs are also associated with the degradation of the epidermal basement membrane/extracellular matrix and the formation of wrinkles, resulting in skin aging [10]. MMP-9 and MMP-2 potently promote the degradation of collagen type IV, a major component of the basement membrane.

Basement membranes are specialized, sheet-like extracellular matrixes that divide tissues into two compartments, i.e., the epithelia and the stromal tissue. The basement membranes located at the dermal-epidermal junction have many functions, the most obvious of which is to tightly link the epidermis to the dermis. The basement membrane is divided into three layers on the basis of morphological studies: the lamina lucida, the lamina densa and the lamina fibroreticularis derived from the epithelia [11]. The lamina densa is a sheet-like structure that is composed mainly of type IV collagen.

Cytokine macrophage migration inhibitory factor (MIF) was first discovered 45 years ago as a T-cell-derived factor that inhibits the random migration of macrophages [12], [13]. MIF was later reevaluated to be a proinflammatory cytokine and pituitary-derived hormone that potentiates endotoxemia [14], [15]. Subsequent work showed that T-cells and macrophages secrete MIF in response to glucocorticoids as well as activation by various proinflammatory stimuli [16]. MIF functions as a pleiotropic cytokine by participating in inflammation and immune responses [17]. MIF is overexpressed in many solid tumors [18], [19]. In many cases, the degree of MIF overexpression is correlated with tumor progression and/or metastatic potential. MIF plays a key role in cell proliferation and angiogenesis [20], [21]. MIF is expressed primarily in T-cells and macrophages; however, recent studies have revealed that this protein is ubiquitously expressed by various types of cells [22]–[25]. MIF is expressed in the epidermis of skin, particularly in the basal layer [26], and plays a critical role in numerous inflammatory skin diseases [27], [28]. Skin keratinocytes are capable of producing a variety of cytokines and are thought to be the principal source of cytokines derived from the epidermis after UV irradiation. Enhanced MIF production is observed in the skin after UVB irradiation [29]. Therefore, MIF may play a pathophysiological role in inflammatory reactions in the skin.

This study investigated the role of MIF in UVB-induced basement membrane damage using MIF transgenic (MIF Tg) mice. Furthermore, the expressions of MMPs were examined in cultured keratinocytes and fibroblasts obtained from MIF Tg mice.

## Materials and Methods

### Reagents

The following materials were obtained from commercial sources. The Isogen RNA extraction kit was purchased from Nippon Gene (Tokyo, Japan), M-MLV reverse transcriptase was purchased from GIBCO (Grand Island, NY), Taq DNA polymerase was purchased from Perkin-Elmer (Norwalk, CO), nylon membranes were purchased from Schleicher & Schuell (Keene, NH), Dulbecco's modified Eagle's minimal medium (DMEM) was purchased from Gibco (Grand Island, NY) and CnT-07 was purchased from CellnTec Co (Huissen, The Netherlands). Recombinant rat MIF (which cross-reacts with that of mice [30]) was expressed in *Escherichia coli* BL21/DE3 (Novagen, Madison, WI) and was purified as described previously [31]. The protein eluate was dialyzed extensively against phosphate buffer saline (PBS) to remove reduced glutathione, passed over a polymyxin B column (Pierce, Rockford, IL), or subjected to Triton X-114 extraction to remove any contaminating lipopolysaccharide, and was stored in aliquots at −20°C until use. MIF siRNA (sc-37138), anti-MMP-2 and MMP-9 polyclonal antibodies were purchased from Santa Cruz Biotechnology (Santa Cruz, CA), anti-type IV collagen polyclonal antibodies were purchased from Abcam (Cambridge, MA), casein and anti-β-actin antibodies were purchased from Sigma-Aldrich Co (St. Louis, MO). The Western blot detection system was obtained from Cell Signaling Technology (Beverly, MA). The MIF inhibitor (ISO-1) was obtained from Merck (Darmstadt, Germany). All other reagents were of analytical grade.

### UVB radiation

The UVB light source was a BLE-8T312 bulb (Spectronics Corp., Westbury, NY) fluorescent lamp that emitted 0.1 mW/cm^2^ of UV between 280 and 315 nm (peak 306 nm) at a distance of 40 cm, as measured by a UV radiometer (UVP, Inc., Upland, CA).

### Mice and treatments

MIF-overexpressing transgenic (MIF Tg) mice were established following cDNA microinjection. The physical and biochemical characteristics of mice, including their body weight (30 g approx.), blood pressure, blood sugar and the serum levels of cholesterol were normal, as reported previously [32]. The expression of the transgene was regulated by a hybrid promoter composed of a cytomegalovirus (CMV) enhancer and a β-actin/β-globin promoter, as reported previously [33]. The strain of the original MIF Tg was ICR, and the mice were backcrossed with C57BL/6 for at least 10 generations. The Tg mice were maintained using heterozygous sibling mating. MIF Tg and wild-type (WT) mice were maintained under specific pathogen-free conditions at the Institute for Animal Experiments at the University of Toyama. The experiments using mice were conducted according to the guidelines set by the University of Toyama Institutional Animal Care and Use Committee under an approved protocol. All experiments were performed on 8-week-old female adult mice (Five mice were used in each group). The MIF Tg and WT mice had their backs shaved with electric clippers and were exposed to 300 mJ/cm^2^ of UVB. UVB radiation was administered three times weekly (on days 1, 3 and 5), and skin was obtained at 0, 2, 4 and 10 weeks. Following UVB irradiation, the mice were euthanized at the indicated time points. In brief, all mice were injected intraperitoneally with pentobarbital (10%) at 10 mg/kg, including control mice, and we waited for five minutes before macroscopically observing the mice and excising skin sections from the dorsal surface, which were used for the RT-PCR, Western blot, zymography, electron microscopy or immunohistochemical staining studies. The experiments were repeated two times.

### Ethics Statement

This study was carried out in strict accordance with the recommendations in the Institute for Animal Experiments at University of Toyama. The protocol was approved by the Committee on the Ethics of Animal Experiments of University of Toyama.

### Reverse transcription-PCR (RT-PCR) analysis

Total RNA was extracted from each murine skin specimen or cultured cells. RNA reverse transcription was performed with M-MLV reverse transcriptase using random hexamer primers and subsequent amplification using Taq DNA polymerase. PCR was carried out for 35 cycles for MMP-2 and MMP-9 and for 40 cycles for MIF, with denaturation at 94°C for 30 seconds, annealing from 46 to 64°C for one minute and extension at 72°C for 45 seconds using a thermal cycler (PE Applied Biosystems Gene Amp PCR system 9700) [34]. The murine MIF primers used in the present study were 5′-GTTTCTGTCGGAGCTCAC-3′ (forward) and 5′-AGCGAAGGTGGAACCGTTCCA-3′ (reverse). The MMP-2 primers used were: 5′-AGATCTTCTTCTTCAAGGACCGGTT-3′ (forward) and 5′-GGCTGGTCAGTGGCTTGGGGTA-3′ (reverse). The MMP-9 primers used were: 5′-CGACAGCACCTCCCACTATG-3′ (forward) and 5′-CCCAACTTATCCAGACTCCT-3′ (reverse) [35]. After PCR, the amplified products were analyzed using 2% agarose gel electrophoresis.

### Western blot analysis

The each murine skin specimen was homogenized with a Polytron homogenizer (Kinematica, Lausanne, Switzerland). Homogenized skin or cultured cells were collected and washed with cold PBS then lysed in RIPA buffer (1 M Tris-HCl, 5 M NaCl, 1% Nonidet P-40 (v/v), 1% sodium deoxycholate, 0.05% SDS, 1 mM phenylmethyl sulfonyl fluoride) for 20 minutes. Following brief sonication, the lysates were centrifuged at 12,000×g for 10 minutes at 4°C, and the protein content in the supernatant was measured using the Bio-Rad protein assay kit (Bio-Rad, Hercules, CA). The protein lysates were denatured at 96°C for 5 minutes after mixing with 5 μl of SDS-loading buffer, applied on an SDS-polyacrylamide gel for electrophoresis and transferred to nitrocellulose membranes. A Western blot analysis was performed to detect the MIF, MMP-2, MMP-9 and type IV collagen expressions using specific antibodies. The band signals were visualized on X-ray film using a chemiluminescence ECL detection reagent (Amersham Biosciences, Buckinghamshire, UK) [36]. The band density was quantified using a BIO-ID image analyzer, and the relative amounts of proteins associated with specific antibodies were normalized according to the intensities of β-actin.

### In situ gelatin zymography

The gelatinolytic activities of tryptase-activated MMP-2 and MMP-9 were analyzed using zymography according to the method of Heussen and Dowle [37], with some modifications. MMP-2 and MMP-9 exist predominantly in the zymogen (proMMP-2 and proMMP-9) form, with a small amount of active enzyme present. The skin samples were placed on 12% acrylamide gel containing 0.1% human type IV collagen (Sigma-Aldrich) under non-reducing conditions. Electrophoresis was carried out at 4°C. After electrophoresis, the gel was incubated with zymogram renaturing buffer for 30 minutes. Then, the gel was transferred to zymogram developing buffer and incubated overnight at 37°C then stained with Coomassie brilliant blue 250-R (CBB). The zymograms were destained and visualized as transparent areas against a blue background.

### Electron microscopy

The dorsal skin was collected and fixed with Karnovsky fixative in 0.1 M cacodylate buffer for two hours and 1% osmium tetroxide for one hour at 41°C. The tissues were dehydrated through a graded ethanol series and embedded in Epon 812. Ultrathin sections were cut using an ultramicrotome (Reichert Ultracut S, Reichert, Vienna, Austria), stained with saturated uranyl acetate and lead citrate and observed under a transmission electron microscope (Hitachi H-7100, Hitachi Co. Ltd., Tokyo, Japan). The disruptions area of the basal lamina were counted under electron microscope at a magnification of ×12000 and expressed as the mean number in ten random fields (two sections per mouse, five mice per group).

### Immunohistochemical staining

Six micrometer-thick skin sections were stained with hematoxylin and eosin (H&E). Neutrophils were counted under a microscope at a magnification of ×400 and expressed as the mean number of cells in five random fields (one section per mouse, five mice per group). Mast cells were detected using classical staining of sulfated proteoglycans in secretory granules. The tissue sections were dewaxed, rehydrated and immersed in 0.1% toluidine blue (Sigma-Aldrich) in 1% NaCl for one to two minutes. The slides were then rinsed in distilled water, dehydrated and mounted.

### MIF siRNA transfection

A total of 2×10^5^ cells were plated in a 6-well plate for 24 hours, and MIF siRNA transfection was conducted using the Lipofectamine 2000 kit according to the manufacturer's instructions (Invitrogen, Carlsbad, CA). We found that at concentrations greater than 30 pmol/ml siRNA treatment was toxic to cells and the extent of cell death increased dose-dependently with increasing siRNA concentrations (data not shown). Therefore, we selected a dose of 30 pmol/ml for optimal transfection of cells with MIF siRNA and then performed the subsequent experiments.

### Cell culture and treatments

Skin specimens were obtained from the dorsal surfaces of newborn MIF Tg and WT mice. The skin specimens were cut into 3 to 5 mm pieces and placed on a large petri dish with the subcutaneous side down, followed by tissue incubation for one week in a humidified atmosphere of 5% CO_2_ at 37°C. These skin specimens were floated on a 0.25% solution of dispase in PBS at 4°C. The time of protease incubation was varied from 24 hours. Epidermis was separated from the dermis with a pair of forceps, and both were washed three times in PBS. Keratinocytes were grown in CnT-07 culture medium containing a low calcium concentration (0.07 mM) and were passaged by trypsin digestion [38]. Once sufficient numbers of fibroblasts had migrated out of the dermal sections in DMEM containing 10% fetal calf serum and 1% penicillin/streptomycin, the pieces of skin were removed, and fibroblasts were cultured in DMEM and passaged by trypsin digestion [39]. The pure keratinocytes and fibroblasts from passage 1 were used for the subsequent experiments. Cells (approximately 80% confluent) were washed with PBS and exposed to UVB light (30 mJ/cm^2^) in PBS. After irradiation, the keratinocytes were cultured in fresh CnT-07 culture medium, and the fibroblasts were cultured in fresh DMEM at 37°C for six or 24 hours. Then, the cells were analyzed by RT-PCR or a Western blot analysis. In some experiments, the cells from WT mice were pretreated with ISO-1 at 50 μM for 24 hours or MIF siRNA (30 pmol/ml) for 48 hours, and then were exposed to UVB light (30 mJ/cm^2^).

### Isolation of neutrophils from the peritoneum and treatment

Murine neutrophils were obtained by abdominal lavage from WT mice by casein stimulation [40]. In brief, WT mice were injected intraperitoneally with 5 ml of 1% casein. Mice were sacrificed 24 hours post casein injection and total intraperitoneal cells were obtained by injecting 10 ml of ice-cold PBS containing 1 mM EDTA into the peritoneal cavity. After gentle massage, the fluid was harvested and the cells were centrifuged and washed before further use. The purity of neutrophils was approximately 90%, as determined by May-Grunwald-Giemsa staining. The neutrophils were pretreated with recombinant MIF at different concentrations (10, 50 and 100 μM) for six or 24 hours, and cells were analyzed by RT-PCR or a Western blot analysis.

### Statistical analysis

All values are expressed as the means ± SD of the respective test or control group. Statistical significance between the control and test groups was evaluated using either Student's t-test or one-way ANOVA. Values of p<0.05 were considered to be significant.

## Results

### Enhanced expression and production of MIF in MIF Tg mice skin following chronic UVB exposure

**Figure 1:**
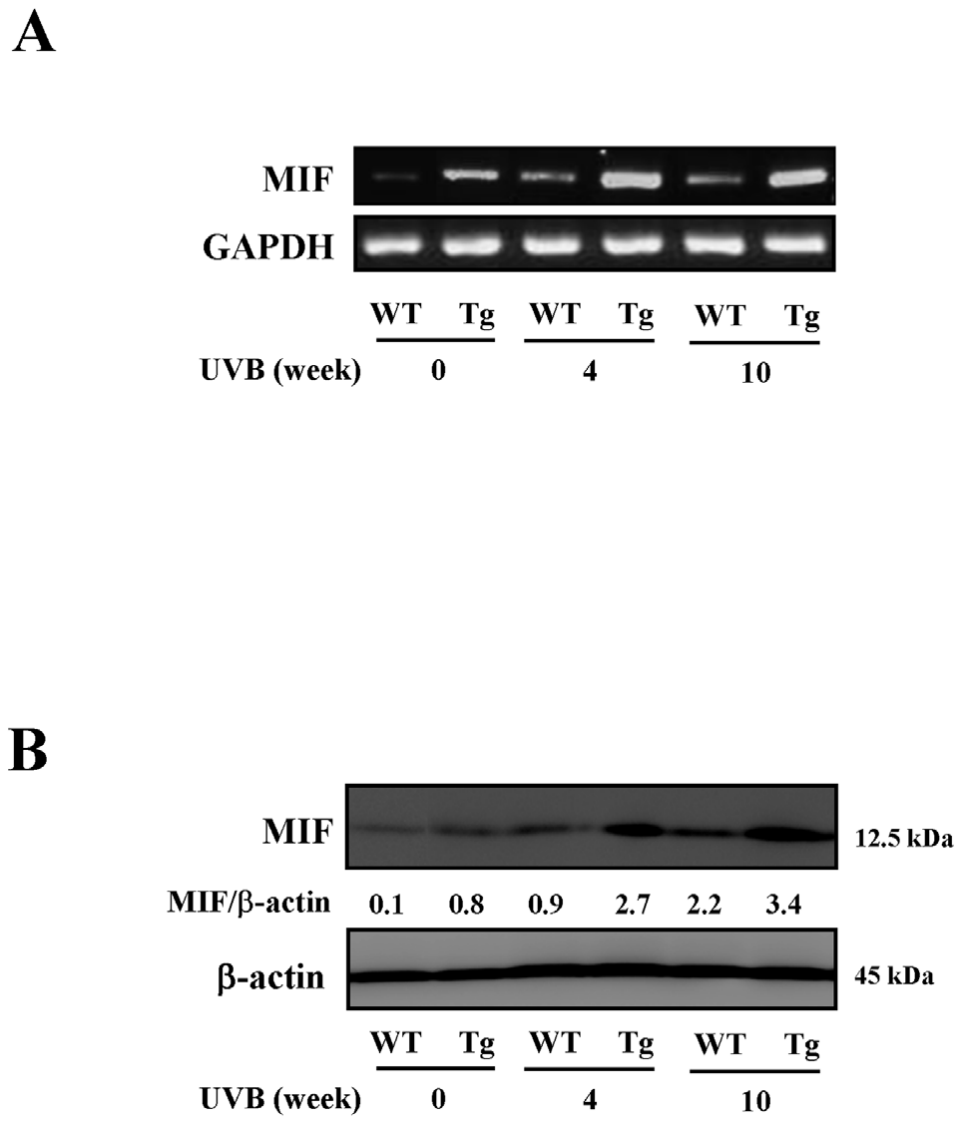
Enhanced expression and production of MIF in the MIF Tg mouse skin following chronic UVB exposure. The total RNA and protein were isolated at 0, 4 and 10(300 mJ/cm^2^×3/week). (A) The expression of MIF mRNA was examined by RT-PCR. (B) A Western blot analysis was performed, and β-actin was used to normalize each expression level. A densitometric analysis was also performed to quantify the intensity of each band. (Each experiment was repeated two times with similar results).

We first examined the expression of MIF mRNA and production of MIF protein in skin specimens excised from MIF Tg and WT mice following chronic exposure to UVB (300 mJ/cm^2^). The mRNA and protein levels of MIF were increased over time in the UVB-irradiated skin in the MIF Tg mice in comparison to the levels observed in the WT mice (Figure 1).

### Enhanced production of MIF, MMP-2 and MMP-9 in MIF Tg mice skin following chronic UVB exposure

**Figure 2:**
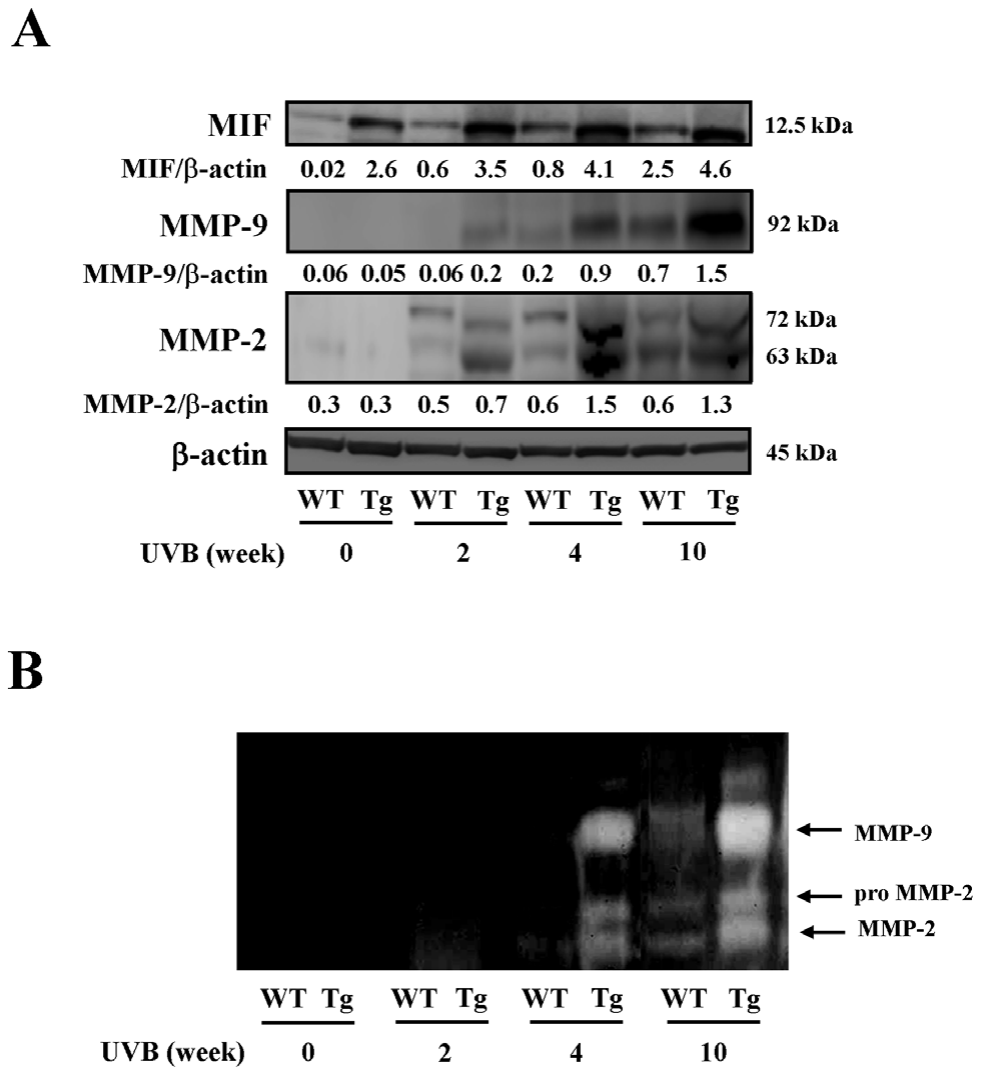
Enhanced production of MIF, MMP-9 and MMP-2 in the MIF Tg mouse skin following chronic UVB exposure. (A) Western blot analysis of MIF, MMP-2 and MMP-9 was conducted to evaluate the effects of UVB (300 mJ/cm^2^×3/week) on the expressions of these compounds in comparison with those observed in WT mice. The band of β-actin was used to normalize each expression level. Each band shows a representative result of the Western blotting analyses, which were repeated five times. A densitometric analysis was also performed to evaluate the intensity of each band. (B) Zymography, using the total skin specimens of each indicative sample, is shown. (These experiments were repeated two times with similar results).

Specimens of irradiated mouse skin were obtained at weeks 0, 2, 4 and 10 for Western blot analyses with antibodies against MIF, MMP-9 and MMP-2. The blots showed that the expressions of MIF, MMP-9 and MMP-2 were increased by chronic UVB exposure in both types of mice. The increases observed were generally time–dependent; however, the MIF Tg mice exhibited higher levels of all specified proteins compared to that observed in the WT mice (Figure 2A). In support of this finding, zymography of the skin specimens demonstrated higher expressions of MMP-9 and MMP-2 proteins at weeks 4 and 10 in the MIF Tg mice (Figure 2B).

### Diminished basement membrane collagen in MIF Tg mice skin following chronic UVB exposure

**Figure 3:**
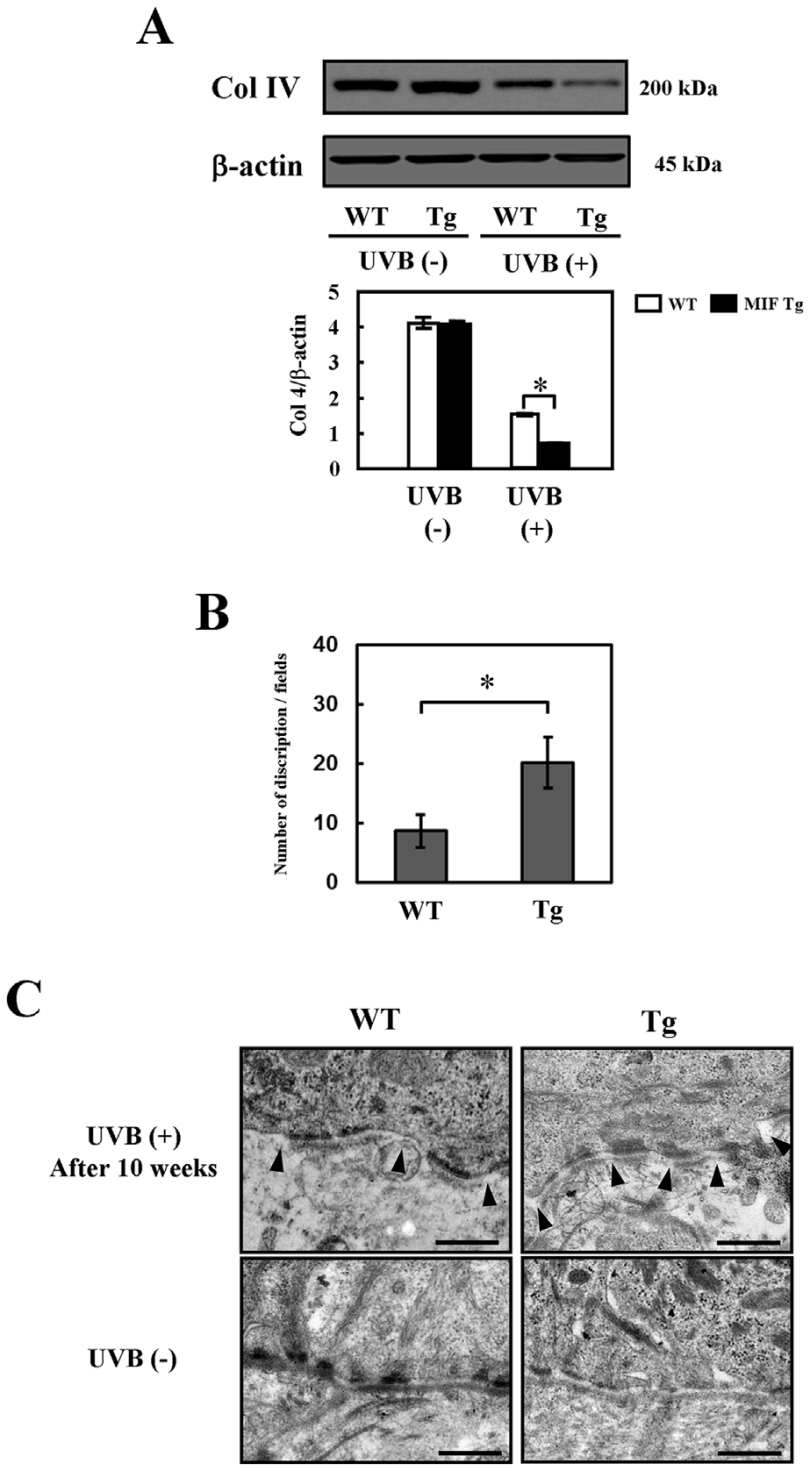
Changes in the levels of basement membrane collagen and basement membrane ultrastructure in the MIF Tg mouse skin following chronic UVB exposure. (A) Western blot analysis of type IV collagen was conducted to evaluate the effects of UVB (300 mJ/cm^2^×3/week) on the expression of this compound. The band of β-actin was used to normalize the expression level. Each band shows a representative result of the Western blotting analyses, which were repeated five times. A densitometric analysis was also performed to evaluate the intensity of each band. *P<0.001. The values reported here represent the means ± SD as determined by a one-way ANOVA. (B) Murine skin was irradiated with UVB (300 mJ/cm^2^×3/week) for 10 weeks. The disruption areas of basal lamina were counted under electron microscope at a magnification of ×12000 and expressed as the mean number in ten random fields (two sections per mouse, five mice per group). *P<0.005. The values reported here represent the means ± SD analyzed by Student's *t-*test. (C) An ultrastructure of junctional cleavages with disruptions of basal lamina in WT mice (a) and MIF Tg mice (b) after 10 weeks of UVB irradiation. An ultrastructure of the normal basal lamina in non-irradiated WT mice (c) and MIF Tg mice (d). Arrowheads indicate the disruptions of basal lamina. Scale bars  = 0.5 μm. (These experiments were repeated two times with similar results).

Murine skin was obtained at weeks 0 and 10 following chronic UVB exposure for Western blot analyses by using type IV collagen antibodies. The data revealed that the level of type IV collagen was decreased in the MIF Tg mice in comparison to that observed in the WT mice (Figure 3A). The relative amounts of proteins associated with specific antibodies were normalized to the band intensities of β-actin.

### Changes in the basement membrane ultrastructure in UVB-radiated MIF Tg and WT mice

In ultrastructure analysis, increased areas of disruption of the basal lamina were observed in the skin of the MIF Tg mice after 10 weeks of UVB irradiation. In contrast, disruptions areas of the basal lamina were not strong in the WT mice compared to those observed in the MIF Tg mice at week 10 after UVB irradiation (p<0.005) (Figure 3B). Representative examples of electron micrographs of the skin samples excised after 10 weeks from UVB irradiation are shown in Figure 3C. Non-irradiated control skin showed normal basal lamina in MIF Tg and WT mice.

### Increased numbers of neutrophils in MIF Tg mice skin following chronic UVB exposure

**Figure 4:**
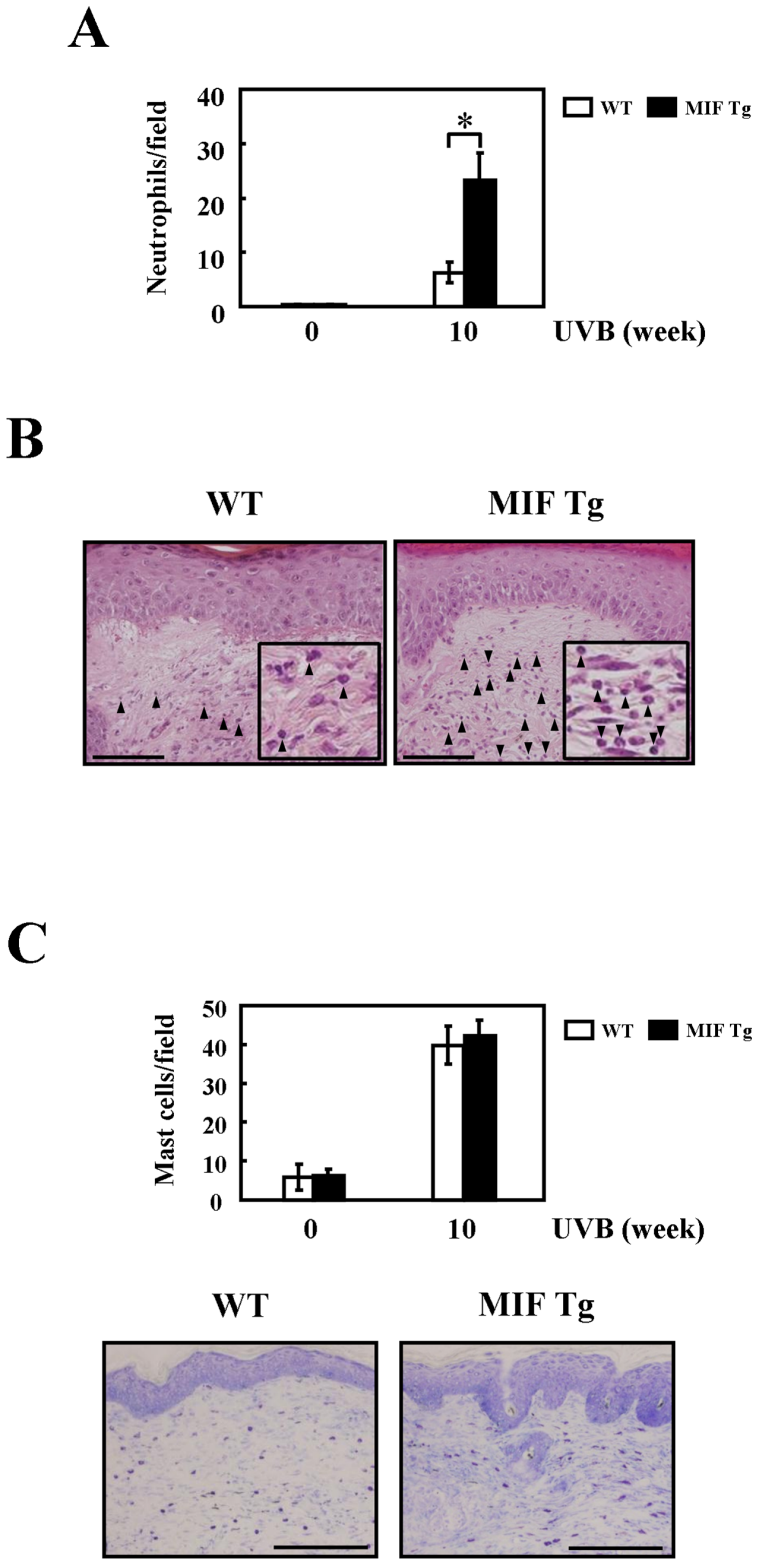
Effects of chronic UVB irradiation on murine skin neutrophils and mast cells. Murine skin was irradiated with UVB (300 mJ/cm^2^×3/week) for 10 weeks. Staining of neutrophils and mast cells in the UV irradiated murine skin sections using the H&E and toluidine blue methods. (A) A quantitative analysis of the number of neutrophils in the UV irradiated (0 and 10 weeks) skin sections was analyzed by a one-way ANOVA (n = 5 in each section). (B) UV irradiated skin (H&E, WT and MIF Tg mouse, 10 weeks). Arrowheads point to neutrophils. Scale bars  = 100 μm. (C) A quantitative analysis of the number of mast cells in the UV irradiated (0 and 10 weeks) skin sections was analyzed by a one-way ANOVA (n = 5 in each section). And UV irradiated skin (Toluidine blue, WT and MIF Tg mouse, 10 weeks). Scale bars  = 200 μm. (The experiments were repeated two times and similar results were obtained).

Murine skin was obtained at weeks 0 and 10 and stained with H&E and toluidine blue to visualize neutrophils and mast cells. The number of neutrophils was increased in the UVB-exposed skin in both types of mice. The MIF Tg mice exhibited a significant increase in the number of neutrophils compared to the WT mice (Figure 4A and B). In addition, the number of mast cells increased following UVB exposure; however, there were no significant differences between the two types of mice (Figure 4C).

### Effects of acute UVB exposure on the MIF expression and production of MMP-9 and MMP-2 in keratinocytes and fibroblasts

**Figure 5:**
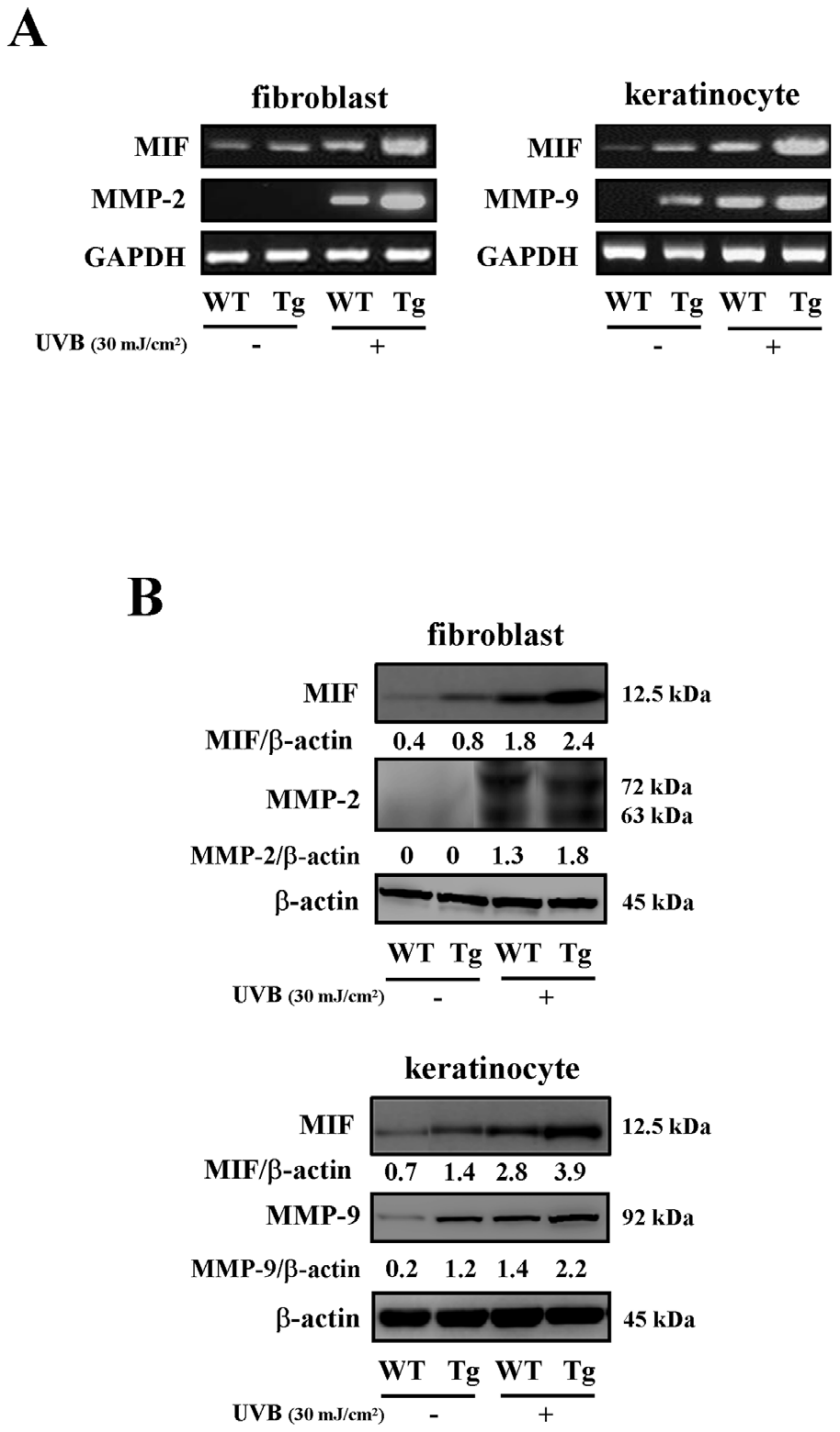
Enhanced expression and production of MMP-9 and MMP-2 in the keratinocytes and fibroblasts of the MIF Tg mice following acute UVB exposure. (A) The expressions of MIF, MMP-2 and MMP-9 mRNA were examined. Total RNA was isolated at six hours after UVB irradiation (30 mJ/cm^2^) and analyzed with RT-PCR (n = 5). (B) Western blot analysis of MIF, MMP-2 and MMP-9 was performed 24 hours after UVB (30 mJ/cm^2^) on the production of these compounds. The band of β-actin was used to normalize the expression level. A densitometric analysis was also performed to evaluate the intensity of each band. The data shown are representative of three independent experiments.

The acute UVB-induced mRNA and protein expression of MIF was intensely increased in the keratinocytes and fibroblasts of the MIF Tg mice (Figure 5). In addition, the mRNA levels of MMP-9 in keratinocytes and MMP-2 in fibroblasts were increased in response to UVB exposure six hours post irradiation in comparison to those observed in the WT mice (Figure 5A). A Western blot analysis revealed that the MMP-9 and MMP-2 expression levels were increased in the UVB-irradiated cells of the MIF Tg mice in comparison to those observed in the WT mice (Figure 5B).

### Inhibitory effects of an MIF inhibitor or MIF siRNA on the acute UVB-induced production of MMP-9 and MMP-2 in keratinocytes and fibroblasts

**Figure 6:**
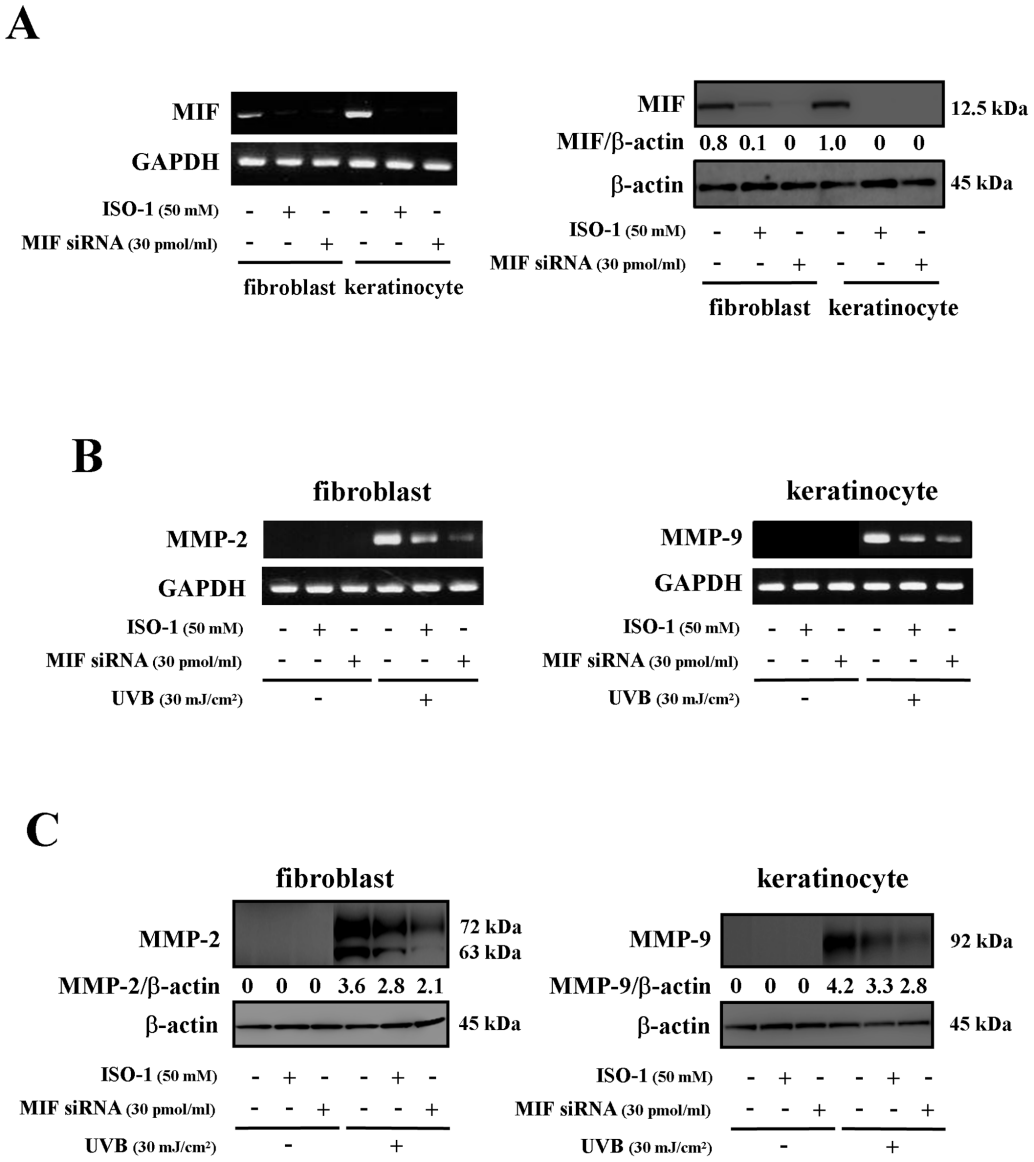
Effects of an MIF inhibitor (ISO-1) or MIF si RNA on the expression and production of MMP-9 and MMP-2 following UVB radiation. (A) The cells were treated with ISO-1 at 50 μM for 24 hours or MIF siRNA (30 pmol/ml) for 48 hours. Next, the expression of MIF mRNA and MIF protein was examined. (B) The expression levels of MMP-9 and MMP-2 mRNA were examined. Cells were pretreated with ISO-1 at 50 μM for 24 hours or MIF siRNA (30 pmol/ml) for 48 hours, and then were exposed to UVB light (30 mJ/cm^2^). Total RNA was isolated six hours after UVB exposure and analyzed by RT-PCR. (C) Cells were pretreated with ISO-1 at 50 μM for 24 hours or MIF siRNA (30 pmol/ml) for 48 hours, and were then exposed to UVB light (30 mJ/cm^2^). The Western blot analysis for the MMP-9 and MMP-2 proteins (30 μg of protein for each group) was performed 24 hours after UVB irradiation. The band of β-actin was used to normalize the expression level. A densitometric analysis was also performed to evaluate the intensity of each band. The data shown are representative of three independent experiments.

To confirm that the presence of MIF was related to the enhancement of acute UVB-induced production of MMP-9 and MMP-2, we examined the effects of ISO-1, an MIF inhibitor, on the MIF levels and UVB-induced production of MMP-9 and MMP-2 in the keratinocytes and fibroblasts of the WT mice. ISO-1 addition resulted in a decrease in the MIF expression in both cell types (Figure 6A). ISO-1 also decreased the UVB-induced mRNA levels and the production of MMP-9 in keratinocytes and MMP-2 in fibroblasts (Figure 6B and C). In addition, we found that the expression of MIF mRNA and protein was significantly reduced in the cells transfected with MIF siRNAs (Figure 6A). The cells treated with MIF siRNA exhibited decreased UVB-induced mRNA levels, and reduced production of MMP-9 in keratinocytes and MMP-2 in fibroblasts (Figures 6B and C).

### Effects of MIF on the acute UVB-induced expression and production of MMP-9 in neutrophils

**Figure 7:**
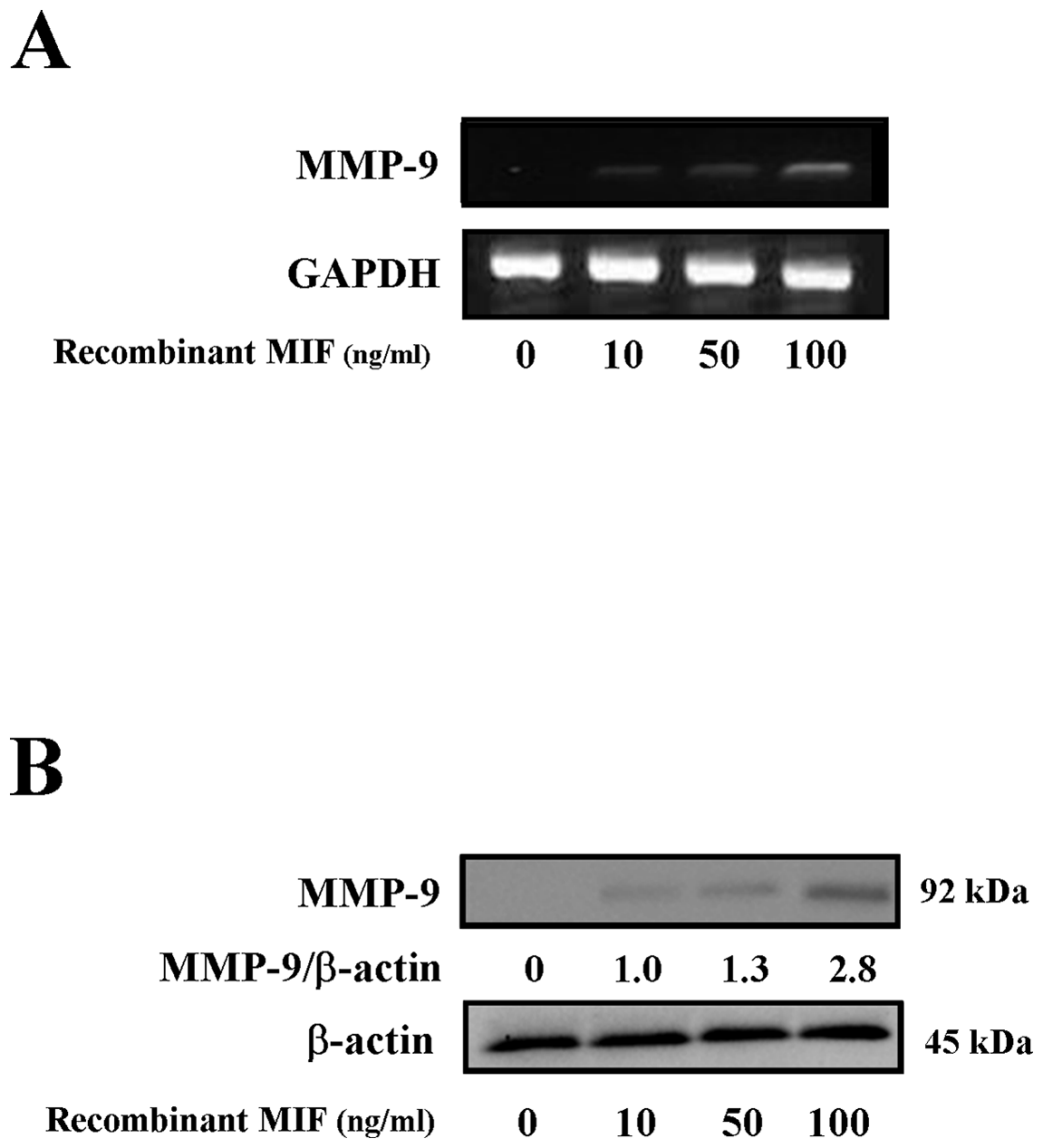
Effects of MIF on the acute UVB-induced expression and production of MMP-9 in neutrophils. Neutrophils (approximately 90% purified) were pretreated with recombinant MIF at different concentrations (10, 50 and 100 μM) for six or 24 hours. (A) Cells were analyzed with RT-PCR of MMP-9. (B) Western blot analysis of the MMP-9 protein expression was then performed. The bands were quantified densitometrically and normalized to the level of β-actin. These experiments were repeated three times with similar results.

To confirm that the MIF was involved in the enhancement of the MMP-9 production induced by the other inflammatory cells, we examined the effects of recombinant MIF on the expression and production of MMP-9 in the neutrophils of the WT mice. As shown in Figure 7, recombinant MIF addition resulted in a dose-dependent increase in the MMP-9 expression and production.

## Discussion

UVB radiation penetrates the epidermis and the upper part of the dermis. The basement membrane at the dermal-epidermal junction is also damaged during the wrinkle formation process. The present study demonstrated that there is a decrease in intact protein levels of type IV collagen and increased basement membrane damage in the exposed skin of MIF Tg mice following chronic UVB exposure compared with that observed in WT mice. The ultra-structural studies showed that significant junctional cleavages with disruption of the basal lamina were observed at the dermal-epidermal junction in MIF Tg mice compared with that observed in WT mice. Consistent with our observations, previous studies have found that detachment of the epidermis from the basal lamina and disruption of the continuity of the basal lamina are the primary ultra-structural findings observed following chronic UVB irradiation in mice [41]. Reduction of collagen IV at the dermal-epidermal junction has also been reported in pronounced wrinkling skin [42].

Various types of UV-induced matrix-degenerating metalloproteinases, which are present in keratinocytes and dermal fibroblasts, have been reported to contribute to the breakdown of dermal interstitial collagen and other connective tissue components. The underlying biological mechanisms causing this skin damage involve a number of secreted cytokines, including IL-1, IL-6 and TNF-α [43]–[45]. UV irradiation upregulates the production of these cytokines, and the production of UV-induced collagenases such as MMP-1 derived from dermal fibroblasts is mediated in part by an IL-1β autocrine mechanism [44]. We have previously shown that MIF upregulates MMP-1 mRNA and MMP-1 activity in dermal fibroblasts. Furthermore, MIF is involved in the upregulation of UVA-induced MMP-1 in dermal fibroblasts through PKC-, PKA-, src-family tyrosine kinase-, MAPK-, c-jun- and AP-1-dependent pathways [46]. IL-1β stimulation leads to a significant increase in the MIF mRNA and protein levels in human dermal fibroblasts [7]. Moreover, it has been reported that UVA irradiation stimulates the production of granzyme B (a serine protease) through MIF [47]. UVA-induced granzyme B degrades dermal fibronectin.

The current study revealed that the skin of MIF Tg mice exhibits higher MIF, MMP-2 and MMP-9 expression levels than those observed in WT mice following chronic UVB exposure. Moreover, the in vitro study found that MIF induces an increase in the MMP-9 expression in cultured keratinocytes and the MMP-2 expression in fibroblasts. Zymogram electrophoresis gels also determined the activities of secreted MMP-9 and MMP-2. Gelatinases MMP-2 and MMP-9 are known to degrade type IV collagen specifically [48], [49] and are components of the epidermal basement membrane. It has been reported that MIF activates the MEK-ERK MAP kinase pathway to induce the MMP-9 expression by murine macrophages [50]. MIF also induces the rheumatoid arthritis synovial fibroblast MMP-2 expression [51]. In support of our observations, it has been reported that several proinflammatory cytokines stimulate the MMP-9 and MMP-2 expressions in keratinocytes and fibroblasts. UVB increases intracellular reactive oxygen species (ROS), which upregulate TGF-β biosynthesis, and activated TGF-β then stimulates the MMP-9 expression in keratinocytes [52]. TNF-α also stimulates the production of MMP-9 in a keratinocyte cell line [53]. Furthermore, TNF-α, IL-1β and IL-6 increase the mRNA and/or protein expression of MMP-2 in fibroblasts [54]. Based on the findings of previous reports and our current evidence, MIF appears to be an important mediator for the production of matrix-degenerating proteases, at least MMPs, which degenerate dermal collagen and basement membrane type IV collagen following UVB or UVA irradiation.

MIF is a cytokine that plays a critical role in several inflammatory conditions. High levels of the MIF expression have been found in a variety of inflammatory conditions, including atopic dermatitis, and inflammatory skin lesions [55]. We also found that chronic UVB exposure in MIF Tg mice results in higher levels of neutrophil infiltration in the dermis compared with that observed in WT mice. Neutrophils are potent cells capable of causing significant tissue damage. They are packed with proteolytic enzymes, including neutrophil elastase and MMPs. Neutrophils have been known to infiltrate the skin following exposure to erythemogenic doses of UVB [56]. Furthermore, activated neutrophils generate and release ROS. Influx of neutrophils can thus damage collagen fibers. Therefore, neutrophils are one of the key players in photoaging, similar to keratinocytes and fibroblasts in the skin, and preventing neutrophil influx following UV radiation appears to be a key factor in the prevention of photoaging. Recent studies have demonstrated that MIF promotes neutrophil trafficking in inflammatory arthritis by facilitating chemokine-induced migratory responses and MAP kinase activation [57]. Our study revealed that cultured neutrophils express and secrete MMP-9 following MIF stimulation in vitro. MIF modulates the functions of human neutrophils by inducing chemokine CXC motif receptor 2(CXCR2)-dependent chemotaxis, enhancing neutrophil survival and promoting the release of chemokine C-C Motif Ligand 4 (CCL4) and MMP-9. Furthermore, neutrophils activated with tumor-derived MIF enhance the migratory properties of HNC cells [58]. In this context, it is speculated that neutrophils that constitutively express MIF are important for mediating basement membrane collagen damage following UVB radiation.

In conclusion, the present study showed that MIF-mediated enhancement of collagen degradation and basement membrane damage occurs primarily through the activation of the MMP-9 and MMP-2 expressions and neutrophil infiltration in the skin following exposure to UVB. Consequently, MIF inhibitors such as ISO-1 may allow for the potential reduction of MMP activation and prevention of neutrophil influx in the skin mediated by UVB.

## References

[1] Ohnishi Y, Tajima S, Akiyama M, Ishibashi A, Kobayashi R, et al (2000) Expression of elastin-related proteins and matrix metalloproteinases in actinic elastosis of sun-damaged skin. Arch Dermatol Res 292: 27–31. 10664012 10.1007/pl00007457

[2] Fisher GJ, Wang ZQ, Datta SC, Varani J, Kang S, et al (1997) Pathophysiology of premature skin aging induced by ultraviolet light. N Engl J Med 337: 1419–1428. 9358139 10.1056/NEJM199711133372003

[3] Brenneisen P, Oh J, Wlaschek M, Wenk J, Briviba K, et al (1996) Ultraviolet B wavelength dependence for the regulation of two major matrix-metalloproteinases and their inhibitor TIMP-1 in human dermal fibroblasts. Photochem Photobiol 64: 877–885. 8931389 10.1111/j.1751-1097.1996.tb01851.x

[4] Goffin L, Seguin-Estevez Q, Alvarez M, Reith W, Chizzolini C (2010) Transcriptional regulation of matrix metalloproteinase-1 and collagen 1A2 explains the anti-fibrotic effect exerted by proteasome inhibition in human dermal fibroblasts. Arthritis Res Ther 12: 1–14. 10.1186/ar2991 PMC2888229 20429888

[5] Lee JS, Park KY, Min HG, Lee SJ, Kim JJ, et al (2010) Negative regulation of stress-induced matrix metalloproteinase-9 by Sirt1 in skin tissue. Exp Dermatol 19: 1060–1066. 20812964 10.1111/j.1600-0625.2010.01129.x

[6] Park HM, Hwang E, Lee KG, Han SM, Cho Y, et al (2011) Royal jelly protects against ultraviolet B-induced photoaging in human skin fibroblasts via enhancing collagen production. J Med Food 14: 899–906. 21812645 10.1089/jmf.2010.1363

[7] Honda A, Abe R, Makino T, Norisugi O, et al (2008) Interleukin-1beta and macrophage migration inhibitory factor (MIF) in dermal fibroblasts mediate UVA induced matrix metalloproteinase-1 expression. J Dermatol Sci 49: 63–72. 18060744 10.1016/j.jdermsci.2007.09.007

[8] Park CH, Chung JH (2011) Epidermal growth factorinduced matrix metalloproteinase-1 expression is negatively regulated by p38 MAPK in human skin fibroblasts. J Dermatol Sci 64: 134–141. 21872446 10.1016/j.jdermsci.2011.07.002

[9] Obayashi K, Kurihara K, Okano Y, Masaki H, et al (2005) L-Ergothioneine scavenges superoxide and singlet oxygen and suppresses TNF-alpha and MMP-1 expression in UVirradiated human dermal fibroblasts. J Cosmet Sci 56: 17–27. 15744438

[10] De Arcangelis A, Neuville P, Boukamel R, Lefebvre O, Kedinger M, et al (1996) Inhibition of laminin alpha 1-chain expression leads to alteration of basement membrane assembly and cell differentiation. J Cell Biol 133: 417–430. 8609173 10.1083/jcb.133.2.417 PMC2120787

[11] Inoue S (1989) Ultrastructure of basement membranes. Int Rev Cytol 117: 57–98. 2684892 10.1016/s0074-7696(08)61334-0

[12] David JR (1996) Delayed hypersensitivity in vitro: its mediation by cell free substances formed by lymphoid cell-antigen interaction. Proc Natl Acad Sci USA 56: 72–77. 10.1073/pnas.56.1.72 PMC285677 5229858

[13] Bloom BR, Bennet B (1966) Mechanism of a reaction in vitro associated with delayed-type hypersensitivity. Science 153: 80–82. 5938421 10.1126/science.153.3731.80

[14] Bernhagen J, Calandra T, Mitchell RA, Martin SB, et al (1993) MIF is a pituitary-derived cytokine that potentiates lethal endotoxaemia. Nature 365: 756–759. 8413654 10.1038/365756a0

[15] Bucala R (1996) MIF re-evaluated: pituitary hormone and glucocorticoid- induced regulator of cytokine production. FASEB J 7: 19–24. 10.1016/1359-6101(96)00008-1 8864351

[16] Calandra T, Bernhagen J, Mitchell RA, Bucala R (1994) The macrophage is an important and previously unrecognized source of macrophage migration inhibitory factor. J Exp Med 179: 895–902. 10.1084/jem.179.6.1895 PMC2191507 8195715

[17] Nishihira J (2000) Macrophage Migration inhibitory factor (MIF): Its essential role in the immune system and cell growth. J Interferon Cytokine Res 20: 751–762. 11032394 10.1089/10799900050151012

[18] Meyer-Siegler K, Hudson PB (1996) Enhanced expression of macrophage migration inhibitory factor in prostatic adenocarcinoma metastases. Urology 48: 448–452. 8804500 10.1016/S0090-4295(96)00207-5

[19] Bando H, Matsumoto G, Bando M, Muta M, Ogawa T, et al (2002) Expression of macrophage migration inhibitory factor in human breast cancer: association with nodal spread. Jpn J Cancer Res 93: 389–396. 11985788 10.1111/j.1349-7006.2002.tb01269.x PMC5927007

[20] Shimizu T, Abe R, Nakamura H, Ohkawara A, et al (1999) High expression of macrophage migration inhibitory factor in human melanoma cells and its role in tumor cell growth and angiogenesis. Biochem Biophys Res Commun 264: 751–758. 10544003 10.1006/bbrc.1999.1584

[21] Chesney J, Metz C, Bacher M, Peng T, Meinhardt A, et al (1999) An essential role for macrophage migration inhibitory factor (MIF) in angiogenesis and the growth of a murine lymphoma. Mol Med 5: 181–191. 10404515 PMC2230298

[22] Lanahan A, Williams JB, Sanders LK, Nathans D (1992) Growth factor induced delayed early response genes. Mol Cell Biol 12: 3919–3929. 1508193 10.1128/mcb.12.9.3919-3929.1992 PMC360271

[23] Wistow GJ, Shaughnessy MP, Lee DC, Hodin J, et al (1993) A macrophage migration inhibitory factor is expressed in the differentiating cells of the eye lens. Proc Natl Acad Sci USA 90: 1272–1275. 7679497 10.1073/pnas.90.4.1272 PMC45854

[24] Bacher M, Meihnardt A, Lan HY, Mu W, Metz CN, et al (1997) Migration inhibitory factor expression in experimentally induced endotoxemia. Am J Pathol 150: 235–246. 9006339 PMC1858503

[25] Nishihira J (2000) Macrophage miration inhibitory factor (MIF): its essential role in the immune system and cell growth. J. Interferon. Cytokine Res 20: 751–762. 10.1089/10799900050151012 11032394

[26] Shimizu T, Ohkawara A, Nishihira J, Sakamoto W (1996) Identification of macrophage migration inhibitory factor (MIF) in human skin and its immmunohistochemical localization. FEBS Lett 381: 199–202. 8601455 10.1016/0014-5793(96)00120-2

[27] Hoi AY, Morand EF, Leech M (2003) Is macrophage migration inhibitory factor a therapeutic target in systemic lupus erythematosus? Immunol. Cell Biol 81: 367–373. 10.1046/j.1440-1711.2003.01183.x 12969324

[28] Foote A, Kipen Y, Santos L, Leech M, et al (2004) Macrophage migration inhibitory factor in systemic lupus erythematosus. J Rheumatol 31: 268–273. 14760795

[29] Shimizu T, Abe R, Ohkawara A, Nishihira J (1999) Ultraviolet B radiation up-regulates the production of macrophage migration inhibitory factor (MIF) in human epidermal keratinocytes. J Invest Dermatol 112: 210–215. 9989798 10.1046/j.1523-1747.1999.00486.x

[30] Takahashi N, Nishihira J, Sato Y, Kondo M, Ogawa H, et al (1998) Involvement of macrophage migration inhibitory factor (MIF) in the mechanism of tumor cell growth. Mol Med 4: 707–714. 9932108 PMC2230345

[31] Nishihira J, Kuriyama T, Nishino H, Ishibashi T, Sakai M, et al (1993) Purification and characterization of human macrophage migration inhibitory factor: evidence for specific binding to glutathione and formation of subunit structure. Biochem Mol Biol Int 31: 841–850. 8136702

[32] Sasaki S, Nishihira J, Ishibashi T, Yamasaki Y, Obikane K, et al (2004) Transgene of MIF induces podocyte injury and progressive mesangial sclerosis in the mouse kidney. Kidney Int 65: 469–481. 14717917 10.1111/j.1523-1755.2004.00394.x

[33] Akagi Y, Isaka Y, Akagi A, Ikawa M, Takenaka M, et al (1997) Transcriptional activation of a hybrid promoter composed of cytomegalovirus enhancer and beta-actin/beta-globin gene in glomerular epithelial cells in vivo. Kidney Int 51: 1265–1269. 9083295 10.1038/ki.1997.172

[34] Yoshihisa Y, Makino T, Matsunaga K, Honda A, Norisugi O, et al (2011) Macrophage migration inhibitory factor is essential for eosinophil recruitment in allergen-induced skin inflammation. J Invest Dermatol 131: 925–931. 21191413 10.1038/jid.2010.418

[35] Yiqin Y, Meilin X, Jie X, Keping Z (2009) Aspirin inhibits MMP-2 and MMP-9 expression and activity through PPARalpha/gamma and TIMP-1-mediated mechanisms in cultured mouse celiac macrophages. Inflammation 32: 233–241. 19462226 10.1007/s10753-009-9125-3

[36] Cui ZG, Kondo T, Ogawa R, Feril LB Jr, Zhao QL, et al (2004) Enhancement of radiation-induced apoptosis by 6-formylpterin. Free Radic Res 38: 363–373. 15190933 10.1080/1071576042000191754

[37] Heussen C, Dowdle EB (1980) Electrophoretic analysis of plasminogen activators in polyacrylamide gels containing sodium dodecyl sulfate and copolymerized substrates. Anal Biochem 102: 196–202. 7188842 10.1016/0003-2697(80)90338-3

[38] Segrelles C, Holguín A, Hernández P, Ariza JM, Paramio JM, et al (2011) Establishment of a murine epidermal cell line suitable for in vitro and in vivo skin modelling. BMC Dermatol 11: 9. 21510892 10.1186/1471-5945-11-9 PMC3113952

[39] Zhao Y, Shimizu T, Nishihira J, Koyama Y, Kushibiki T, et al (2005) Tissue regeneration using macrophage migration inhibitory factor-impregnated gelatin microbeads in cutaneous wounds. Am J Pathol 167: 1519–1529. 16314467 10.1016/S0002-9440(10)61238-2 PMC1613201

[40] Coligan JE. (1994) Current protocols in immunology. John Wiley & Sons, Inc., New York, N.Y.

[41] Inomata S, Matsunaga Y, Amano S, Takada K, Kobayashi K, et al (2003) Possible involvement of gelatinases in basement membrane damage and wrinkle formation in chronically ultraviolet B-exposed hairless mouse. J Invest Dermatol 120: 128–134. 12535209 10.1046/j.1523-1747.2003.12021.x

[42] Contet-Audonneau JL, Jeanmaire C, Pauly G (1999) A histological study of human wrinkle structures: comparison between sun-exposed areas of the face, with or without wrinkles, and sun-protected areas. Br J Dermatol 140: 1038–1047. 10354068 10.1046/j.1365-2133.1999.02901.x

[43] Alexander JP, Acott TS (2001) Involvement of protein kinase C in TNF-α regulation of trabecular matrix metalloproteinases and TIMPs. Invest Ophthalmol Vis Sci 4: 2831–2838. 11687525

[44] Wlaschek M, Heinen G, Poswig A, Schwarz A, et al (1994) UVA-induced autocrine stimulation of fibroblast-derived collagenase/MMP-1 by interrelated loops of interleukin-1 and interleukin-6. Photochem Photobiol 59: 550–556. 8041811 10.1111/j.1751-1097.1994.tb02982.x

[45] Rutter JL, Benbow U, Coon CI, Brinckerhoff CE (1997) Cell-type specific regulation of human interstitial collagenase-1 gene expression by interleukin-1 beta (IL-1 beta) in human fibroblasts and BC-8701 breast cancer cells. J Cell Biochem 66: 322–236. 9257189

[46] Watanabe H, Shimizu T, Nishihira J, Abe R, Nakayama T, et al (2004) Ultraviolet A-induced production of matrix metalloproteinase-1 is mediated by macrophage migration inhibitory factor (MIF) in human dermal fibroblasts. J Biol Chem 279: 1676–1683. 14581488 10.1074/jbc.M303650200

[47] Hernandez-Pigeon H, Jean C, Charruyer A, Haure MJ, et al (2007) UVA induces granzyme B in human keratinocytes through MIF: implication in extracellular matrix remodeling. J Biol Chem 282: 8157–8164. 17224449 10.1074/jbc.M607436200

[48] Seltzer JL, Weingarten H, Akers KT, Eschbach ML, et al (1989) Cleavage specificity of type IV collagenase (gelatinase) from human skin. Use of synthetic peptides as model substrates. J Biol Chem 264: 19583–19586. 2555325

[49] Aimes RT, Quigley JP (1995) Matrix metalloproteinase-2 is an interstitial collagenase. Inhibitor-free enzyme catalyzes the cleavage of collagen fibrils and soluble native type I collagen generating the specific 3/4- and 1/4-length fragments. J Biol Chem 270: 5872–5876. 7890717 10.1074/jbc.270.11.5872

[50] Yu X, Lin SG, Huang XR, Bacher M, Leng L, et al (2007) Macrophage migration inhibitory factor induces MMP-9 expression in macrophages via the MEK-ERK MAP kinase pathway. J Interferon Cytokine Res 27: 103–109. 17316137 10.1089/jir.2006.0054

[51] Pakozdi A, Amin MA, Haas CS, Martinez RJ, Haines GK 3rd, et al (2006) Macrophage migration inhibitory factor: a mediator of matrix metalloproteinase-2 production in rheumatoid arthritis. Arthritis Res Ther 8: R132. 16872482 10.1186/ar2021 PMC1779381

[52] Wang H, Kochevar IE (2005) Involvement of UVB-induced reactive oxygen species in TGF-beta biosynthesis and activation in keratinocytes. Free Radic Biol Med 38: 890–897. 15749385 10.1016/j.freeradbiomed.2004.12.005

[53] Meephansan J, Komine M, Tsuda H, Ohtsuki M (2012) Suppressive effect of calcipotriol on the induction of matrix metalloproteinase (MMP)-9 and MMP-13 in a human squamous cell carcinoma cell line. Clin Exp Dermatol 37: 889–896. 22924547 10.1111/j.1365-2230.2012.04381.x

[54] Wisithphrom K, Windsor LJ (2006) The effects of tumor necrosis factor-alpha, interleukin-1beta, interleukin-6, and transforming growth factor-beta1 on pulp fibroblast mediated collagen degradation. J Endod 32: 853–861. 16934628 10.1016/j.joen.2006.03.017

[55] Nishihira J (1998) Novel pathophysiological aspects of macrophage migration inhibitory factor. Int J Mol Med 2: 17–28. 9854138 10.3892/ijmm.2.1.17

[56] Fisher GJ, Choi HC, Bata-Csorgo Z, Shao Y, Datta S, et al (2001) Ultraviolet irradiation increases matrix metalloproteinase-8 protein in human skin in vivo. J Invest Dermatol 117: 219–226. 11511297 10.1046/j.0022-202x.2001.01432.x

[57] Santos LL, Fan H, Hall P, Ngo D, Mackay CR, et al (2011) Macrophage migration inhibitory factor regulates neutrophil chemotactic responses in inflammatory arthritis in mice. Arthritis Rheum 63: 960–970. 21452319 10.1002/art.30203 PMC3069137

[58] Daryadel A, Grifone RF, Simon HU, Yousefi S (2006) Apoptotic neutrophils release macrophage migration inhibitory factor upon stimulation with tumor necrosis factor-alpha. J Biol Chem 281: 27653–2736261. 16861224 10.1074/jbc.M604051200

